# Usefulness of the IgA and IgG Responses to Macrophage Migration Inhibitory Factor for the Diagnosis of Tuberculosis

**DOI:** 10.3390/diagnostics10110991

**Published:** 2020-11-23

**Authors:** Ji Yeon Lee, Byoung-Jun Kim, Jee-min Kim, Junghyun Kim, Joon-Sung Joh, Ina Jeong, Yoon-Hoh Kook, Bum-Joon Kim

**Affiliations:** 1 Division of Pulmonary and Critical Care Medicine, Department of Internal Medicine, National Medical Center, Seoul 04564, Korea; fulgeo@nmc.or.kr (J.Y.L.); everflying@hanmail.net (J.K.); splendor329@hanmail.net (J.K.); ssabana777@nmc.or.kr (J.-S.J.); inajeong@gmail.com (I.J.); 2 Department of Biomedical Sciences, Microbiology and Immunology and Liver Research Institute, Seoul National University College of Medicine, Seoul 03080, Korea; arukas22@snu.ac.kr

**Keywords:** tuberculosis, serology, diagnosis, biomarkers, macrophage migration inhibitory factor, cytokines

## Abstract

Serological tests offer the potential in order to improve the diagnosis of tuberculosis (TB). Macrophage migration inhibitory factor (MIF) plays a protective role in infection control in TB; however, to date, no studies on antibody responses to MIF have been reported. We measured immunoglobulin (Ig)A and IgG responses to MIF in individuals with either active tuberculosis (ATB; *n* = 65), latent tuberculosis (LTBI; *n* = 53), or in non-infected individuals (NI; *n* = 62). The QuantiFERON-TB Gold In-Tube (QFT-GIT) assay was used in order to screen for LTBI. The level of IgA against MIF was significantly lower in LTBI and ATB patients than in NI individuals, was significantly related to LTBI and ATB diagnosis, and it could discriminate between LTBI and ATB. In contrast, the level of IgG against MIF was significantly lower in LTBI patients than in NI individuals and was significantly related to LTBI diagnosis. Anti-MIF IgG levels were significantly lower in AFB-negative TB, minimal TB, and new ATB patients, than in the NI group. IgA and IgG levels against MIF both showed significant negative correlations with IFN-γ levels, as assessed using the QFT-GIT test. Although none of the antibodies could achieve high diagnostic predictive power individually, our results suggest the possibility of using IgA antibody responses to MIF in the diagnosis of LTBI and ATB.

## 1. Introduction

Tuberculosis (TB) remains a significant threat to public health. In 2018, seven-million new cases of TB were reported worldwide, with 1.2 million TB-related deaths reported among individuals with human immunodeficiency virus (HIV)-negative status, and an additional 251,000 deaths being reported among individuals with HIV-positive status [1]. 

The rapid diagnosis and treatment of TB is essential for its effective management. However, the TB culture test, which is currently the gold standard for TB diagnosis, usually takes 3–8 weeks, leading to delayed diagnosis [2]. Approximately 50% of mycobacterial smears turn out to be negative, and the sensitivity is even lower in extrapulmonary TB [3]. Molecular analysis is costly and it requires the latest technology and high-end laboratory instruments [4].

Because latent tuberculosis infection (LTBI) can progress to active tuberculosis (ATB), its diagnosis and treatment is an important factor in the control of TB infections. However, the interferon-gamma release assay (IGRA), which is currently used to diagnose LTBI, has many limitations. It is not only complex and expensive, but also has poor reproducibility [5,6]. Moreover, it cannot distinguish between LTBI and ATB [7,8].

Serological testing performed while using blood samples has attracted researchers due to its various advantages, which overcome the limitations of existing tests. The serological test is rapid, and it does not require a lesion site specimen, thereby allowing for point-of-care testing. In addition, it is inexpensive and requires little laboratory infrastructure; therefore, it can be conducted, even in resource scarce situations.

Macrophage migration inhibitory factor (MIF) was first identified as a cytokine secreted from activated T cells. It inhibits macrophage migration and enhances their ability to kill intracellular parasites or tumor cells [9,10]. Reportedly, MIF is also secreted from endothelial cells and fibroblasts [11,12], and it is involved in cell growth, T cell activation, and various inflammatory and immune responses [13]. In particular, it induces the synthesis of inflammatory cytokines, and antagonizes the inhibition of cytokine production that is mediated by glucocorticoids [14]. 

Recent studies have shown that MIF expression is increased in TB, as well as in several immune-related diseases. Das et al. reported that, genetically, a lower MIF expression decreases cytokine and reactive oxygen production, thereby inhibiting the killing of mycobacteria and increasing the risk of TB. Thus, MIF seems to play an important, protective role in infection control [15]. 

The presence of naturally occurring autoantibodies against cytokines in healthy individuals and patients has been documented in many reports [16]. Antibody responses to cytokines play important roles in immune regulation [16,17]. Several studies have reported that immunoglobulin (Ig)A and IgG respond to various cytokines, including interferon-gamma (IFN-γ), granulocyte colony-stimulating factor (G-CSF), and tumor necrosis factor-alpha (TNF-α), in *Mycobacterium* infection, HIV infection, alveolar protein syndrome, and autoimmune diseases, with their effects differing, depending on the disease state [18,19,20,21]. When considering the regulatory role of autoantibodies during neutralization, their levels may show a trend opposite to that of cytokines. Therefore, we hypothesized that, in response to increased MIF levels in tuberculosis, antibodies against MIF would be lower in TB patients than in healthy individuals. However, to date, no studies have been conducted on the response of antibodies against MIF in TB.

In the present study, we analyzed the IgA and IgG responses to MIF in the sera of patients with ATB and LTBI, as well as in that of non-infected (NI) individuals. In addition, we investigated whether the antibody responses to MIF are valuable for the rapid diagnosis of TB.

## 2. Materials and Methods

### 2.1. Study Subjects

The participants were enrolled, as described previously [22]. Briefly, 180 individuals, including 65 ATB patients, 53 LTBI patients, and 62 NI individuals, were enrolled at the National Medical Center, Seoul, Republic of Korea. ATB was diagnosed in patients with positive sputum or bronchoscopy specimens, according to a TB nucleic acid amplification test or a mycobacterial culture. For those without chest radiographic abnormalities and clinical symptoms, LTBI was diagnosed based on a positive result from the QuantiFERON-TB Gold In-Tube (QFT-GIT) assay. Individuals were considered to be NI if their QFT-GIT assay results were negative. Five milliliters of peripheral blood were obtained at the time of enrollment. Both laboratory and clinical data were collected during their routine hospital visit. All of the participants were HIV-negative. The median age was 33 years (interquartile range 29–33 years) in the NI group, 49 years (33–57 years) in the LTBI group, and 60 years (51–67 years) in the ATB group. Of the 180 participants, 21 (33.9%) in the ATB group, 21 (39.6%) in the LTBI group, and 56 (86.2%) in the ATB group were male.

### 2.2. Preparation of MIF

Recombinant MIF protein was purified from *Escherichia coli*, as previously described [23,24]. Briefly, BL21 *E. coli* strains (RBC Bioscience, Taipei City, Taiwan) were transformed with pET28a-MIF in order to express and purify the fusion protein. Bacterial cultures were induced with 0.4 mM isopropyl β-d-thiogalactoside (IPTG, Duchefa Biochemie, Haarlem, Netherlands). Next, bacterial cultures were sonicated for 10 min. at 4 °C, and then centrifuged at 1600× *g* for 20 min. at 4 °C. Pellets containing His-tagged MIF (His-MIF) were resuspended in binding buffer (0.5 M NaCl, 20 mM Tris-HCl, 5 mM imidazole) containing 4 M urea (Sigma Aldrich, St. Louis, MO, USA). His-MIF was purified while using Ni-NTA His•Bind Resin (Merck, Darmstadt, Germany) and subsequently eluted using elution buffer (300 mM NaCl, 50 mM sodium phosphate buffer, and 250 mM imidazole) containing 4 M urea. Finally, MIF protein was dialyzed in order to remove imidazole, residual salts, and urea.

### 2.3. Enzyme-Linked Immunosorbent Assay (ELISA)

Antibody isotypes, IgA and IgG, serum levels were assessed while using an ELISA, as previously described with minor modifications [22]. Briefly, Corning 96-well Enzyme Immunoassay/Radio Immunoassay (EIA/RIA) plates (Corning Inc., Kennebunk, ME, USA) were coated overnight at 4 °C with a solution containing 5 μg/mL of MIF diluted in a 0.05 M carbonate-bicarbonate coating buffer. The plates were washed three times with phosphate-buffered saline (PBS) containing 0.05% Tween-20, and subsequently blocked with 5% bovine serum albumin (BSA) in PBS at room temperature (RT) (20–25 °C) for 1 h. Next, 100 μL of the samples diluted (1:10) in PBS were added to each well and then incubated for 2 h at RT. After washing three times with PBS-T buffer (0.05% Tween 20 in PBS), the indicated secondary antibody was diluted to 1:500 in 5% BSA, and 100 μL was added to each well and incubated for 1 h at RT. The following secondary antibodies were used: anti-human IgG (H+L), HRP conjugate (W4038, Promega, Madison, WI), or anti-human IgA HRP-conjugated antibody (PA1-74395, Invitrogen, Rockford, IL, USA). Next, the plates were washed three times with PBS-T buffer, and 100 μL of 3,3′,5,5′-Tetramethylbenzidine (BD OptEIA substrate; BD Biosciences, San Diego, CA, USA) was added to each well. The reaction was quenched after 10 min. using 50 μL of 1 N sulfuric acid. Absorbance was read at 450 nm using an ELISA microplate reader (Tecan Sunrise^TM^; Tecan Group Ltd., Männedorf, Switzerland).

### 2.4. Data Analysis

Antibody levels, presented as OD values, and one-way ANOVA with Bonferroni’s multiple comparisons were used in order to compare IgA and IgG responses among the different groups. We performed binary logistic regression in order to assess the biomarkers ability to predict either LTBI or ATB. The area under the curve (AUC) of the receiver operating characteristics (ROC) curve was calculated to compare each biomarker and its predictive potential. Correlations among IgA and IgG serum levels, in addition to IFN-γ, age, BMI, sex, and drug resistance, were examined while using Pearson’s correlation coefficient. Both SPSS Statistics 17.0 (SPSS Inc., Chicago, IL, USA) and Prism 5.0 (GraphPad Software, La Jolla, CA, USA) were used for data analyses. The results with *p* < 0.05 were regarded statistically significant.

### 2.5. Ethics Statement

All of the subjects provided written, informed consent for inclusion before participating in this study. This study was conducted in accordance with the Declaration of Helsinki, and the protocol was approved by the institutional review board of the National Medical Center (IRB no. H-1811-096-002) and Seoul National University Hospital (H-2006-090-1132).

## 3. Results

### 3.1. Serum Levels of IgA and IgG against MIF

**Figure 1:**
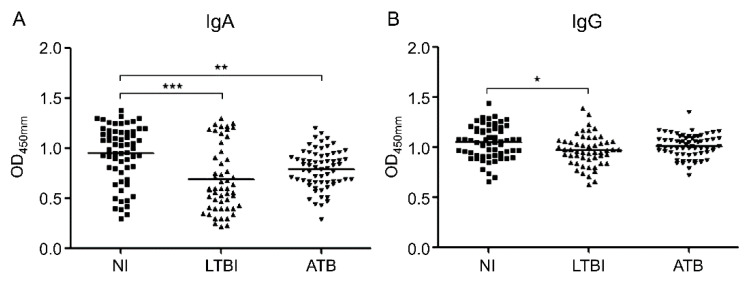
Optical density (OD) values of the anti-macrophage migration inhibitory factor (MIF) immunoglobulin (Ig)A (**A**) and anti-MIF IgG (**B**) serodiagnostic assays for the comparison of 65 active tuberculosis (ATB) patients, 53 latent tuberculosis infection (LTBI) patients, and 62 non-infected (NI) individuals. Each dot represents the values obtained from individuals; horizontal bars indicate the mean values. The groups were compared using one-way ANOVA with Bonferroni’s multiple comparisons. * *p* < 0.05; ** *p* < 0.01; *** *p* < 0.001.

Figure 1 shows the IgA and IgG responses to MIF for each participating group. IgA levels against MIF were significantly lower in the LTBI (*p* < 0.0001) and ATB groups (*p* = 0.006), as compared with the NI group; there was no significant difference between the ATB and LTBI groups (Figure 1A). The anti-MIF IgG levels were significantly lower in the LTBI group, when compared with the NI group (*p* = 0.011). There was no significant difference in IgG levels between the ATB group, and the LTBI and NI groups (Figure 1B). 

The level of IgA against MIF was significantly related to the diagnosis of LTBI (*p <* 0.0001), ATB (*p* = 0.001), as well as to the differential diagnosis of LTBI and ATB (*p* = 0.029), according to the logistic regression analysis. 

**Figure 2:**
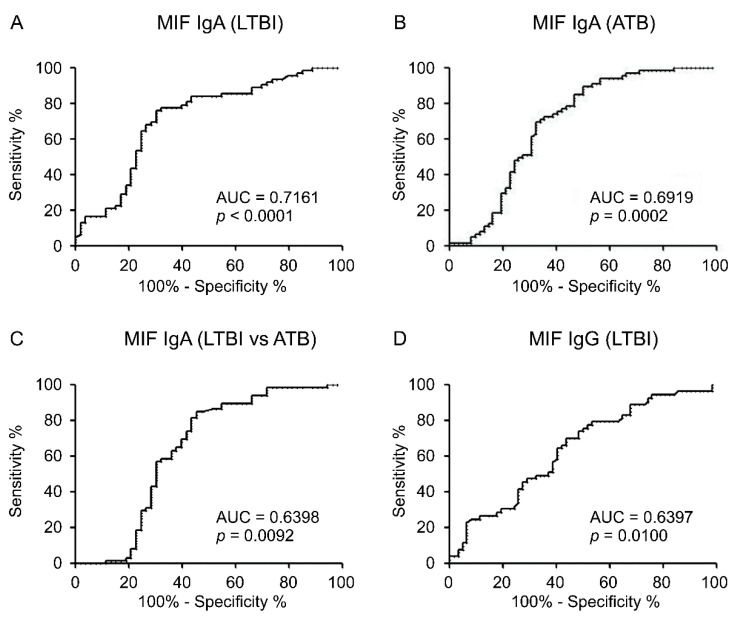
Receiver operating characteristic (ROC) curves were used to evaluate whether anti- macrophage migration inhibitory factor (MIF) immunoglobulin (Ig)A can distinguish between non-infected (NI) and latent tuberculosis infection (LTBI) (**A**), whether anti-MIF IgA can differentiate between NI and active tuberculosis (ATB) (**B**), whether anti-MIF IgA can distinguish LTBI and ATB (**C**), and whether anti-MIF IgG can differentiate between NI and LTBI (**D**). Each area under the curve (AUC) and *p* value are indicated on the graphs.

The AUC for IgA responses to MIF was 0.7161 (*p* < 0.0001) in the diagnosis of LTBI (Figure 2A), 0.6919 *(p* < 0.0002) in the diagnosis of ATB (Figure 2B), and 0.6398 (*p* = 0.0092) in the differential diagnosis of either LTBI or ATB (Figure 2C). The IgG levels against MIF were significantly related to LTBI diagnosis (*p* = 0.011), with an AUC of 0.6397 (*p* = 0.0100) (Figure 2D). However, the IgG response to MIF was not significantly related to ATB diagnosis and differential diagnosis.

Table 1 summarizes the sensitivity and specificity of IgA and IgG against MIF in the diagnosis of ATB and LTBI.

### 3.2. IgA and IgG Serum Levels in Subgroup Analysis

**Figure 3:**
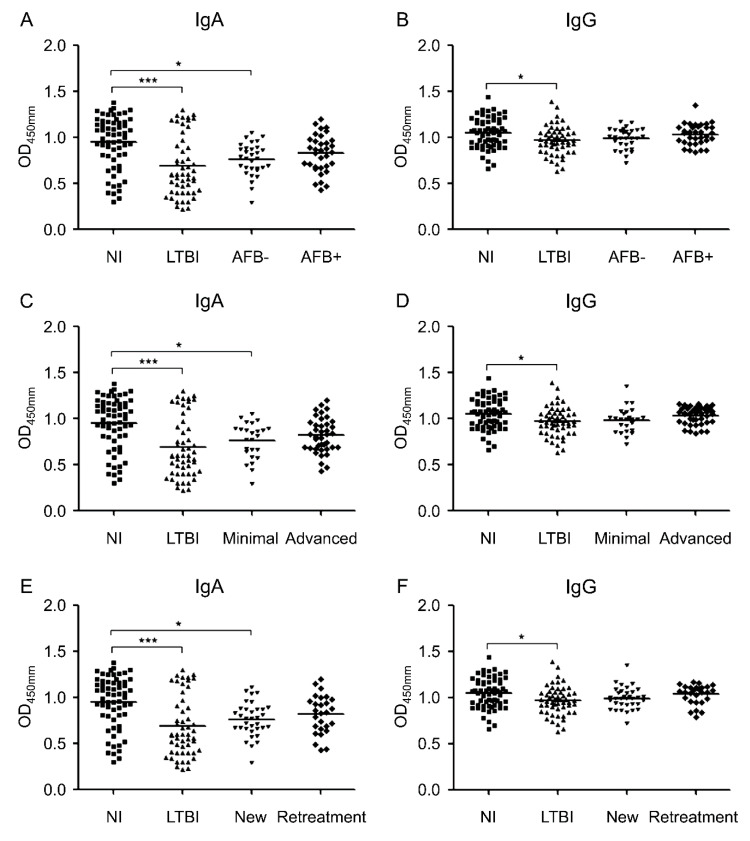
Optical density (OD) values of the anti-macrophage migration inhibitory factor (MIF) immunoglobulin (Ig)A (**A**) and anti-MIF IgG (**B**) serodiagnostic assays for the comparison of 31 acid-fast bacilli (AFB)-negative tuberculosis (AFB-) patients, 34 AFB-positive tuberculosis (AFB+) patients, 53 latent tuberculosis infection (LTBI) patients, and 62 non-infected (NI) individuals. OD values of the anti-MIF IgA (**C**) and anti-MIF IgG (**D**) serodiagnostic assays for the comparison of 25 minimal tuberculosis patients, 40 advanced tuberculosis patients, 53 LTBI patients, and 62 NI individuals. OD values of the anti-MIF IgA (**E**) and anti-MIF IgG (**F**) serodiagnostic assays for the comparison of 34 new patients, 27 retreatment patients, 53 LTBI patients, and 62 NI individuals. Each dot represents the values obtained from individual subjects; horizontal bars indicate the mean values. Groups were compared using one-way ANOVA with Bonferroni’s multiple comparisons. * *p* < 0.05; *** *p* < 0.001.

Figure 3A,B show the IgA and IgG responses to MIF when patients with ATB were classified into either acid-fast bacilli (AFB)-negative or AFB-positive TB groups. The anti-MIF IgA levels were significantly lower in the AFB-negative TB group than in the NI group (*p* = 0.011). However, no significant difference was observed between the NI and AFB-positive TB groups, as well as among the LTBI and both AFB-positive and AFB-negative TB groups (Figure 3A).

The anti-MIF IgG levels showed no significant differences between the LTBI group and both the AFB-negative and AFB-positive TB groups, as well as among the NI group and both AFB-negative and AFB-positive TB groups (Figure 3B).

Moreover, when patients with ATB were categorized into minimal and advanced TB groups, depending on their radiological severity, the IgA levels against MIF were found to be significantly lower in minimal TB groups than in the NI group (*p* = 0.021) (Figure 3C). There was no significant difference in anti-MIF IgG levels between each subgroup of ATB and both the LTBI and NI groups (Figure 3D). The AFB-positive rate was significantly higher at 75.0% (*n* = 30/40) in the advanced TB group when compared with 16.0% (*n* = 4/25) in the minimal TB group (*p* < 0.0001).

Figure 3E and 3F shows the IgA and IgG responses to MIF when the patients with ATB were subdivided either into new patients or retreatment groups. IgA levels against MIF were significantly lower in new patients (*p* = 0.010) than in the NI group (Figure 3E). Further, there was no significant difference in anti-MIF IgG levels when comparing the other groups with both the new patients and retreatment groups (Figure 3F). There was no significant difference in the AFB-positive rate and radiological severity between new patients and retreatment groups.

### 3.3. Correlations Among IgA and IgG Serum Levels, and IFN-γ, Age, BMI, Sex, and Drug Resistance

There was a significant positive correlation between the serum levels of IgA and IgG against MIF (r = 0.564, *p* < 0.0001).

**Figure 4:**
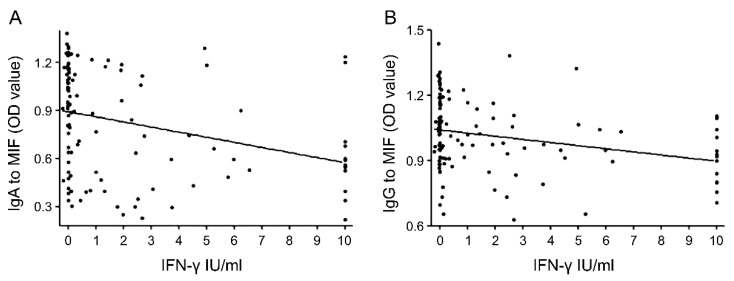
Correlation between the level of interferon-gamma and the levels of serum immunoglobulin (Ig)A (r = −0.325, *p* < 0.0001) (**A**) and IgG (r = −0.295, *p* = 0.001) (**B**) against MIF.

There were trends of a negative correlation between the level of IFN-γ induced in the QFT-GIT test and the OD values of serum IgA (r = −0.325, *p* < 0.0001) and IgG (r = −0.295, *p* = 0.001) against MIF (Figure 4).

Moreover, the serum levels of IgA (r = −0.422, *p* < 0.0001) and IgG (r = −0.254, *p* = 0.001) against MIF had a significant negative correlation with age. In addition, a significant negative correlation was observed between BMI and IgG response against MIF (r = −0.201, *p* = 0.009); no significant correlation existed between BMI and IgA response against MIF (r = −0.111, *p* = 0.154). There were no significant differences in IgA and IgG levels against MIF based on sex and drug resistance.

## 4. Discussion 

In this study, we compared the IgA and IgG responses to MIF in ATB, LTBI, and NI groups. The IgG response against MIF was significantly lower in patients with LTBI than in NI individuals. The anti-MIF IgA levels were significantly lower in both LTBI and ATB patients than in NI individuals. The level of IgA against MIF also showed significant predictive potential in the diagnosis of ATB and LTBI, according to ROC analysis, which suggested that mucosal immunity to MIF may be associated with the pathogenesis of TB. Individually, IgG and IgA antibodies against MIF could not achieve good sensitivity and specificity, respectively, in order to replace conventional diagnostics. In previous studies, higher predictive power could be achieved when tests of multiple markers were combined [25,26,27]. Because statistically significant differences in anti-MIF antibody levels existed between study groups, it may be possible to increase the predictive power of the existing method by combining it with other known markers.

Previous studies have reported increased MIF levels in TB [15,28]. In contrast, the antibody levels against MIF were decreased in TB and LTBI patients as compared to NI individuals, in the present study. We infer that low levels of MIF antibodies may be associated with elevated levels of MIF and macrophage activity in TB, as MIF is expected to modulate the induction or maintenance of Th1 responses in TB [29].

However, the results of this study showed no significant differences in the IgA and IgG levels between ATB and LTBI. In addition, antibody levels against MIF were increased in ATB, advanced TB, and AFB-positive TB patients, when compared to LTBI, minimal TB, and AFB-negative TB patients, respectively, although these increases were not significant.

These results are consistent with previous studies, which reported that circulating MIF levels were not necessarily higher in patients with moderate or far advanced TB, as compared to patients with minimal TB [28]. MIF is secreted from T lymphocytes and macrophages, as well as from anterior pituitary cells in response to stimulation from lipid polysaccharides [30]. Because the level of MIF can be regulated via immune and endocrine systems, it is possible that various regulatory mechanisms are involved in regulating the levels of, and antibodies against, MIF. 

In the present study, both IgA and IgG responses to MIF showed a significant negative correlation with IFN-γ levels. These results suggest that a strategy that is based on IgA and IgG responses against MIF may complement the methods that are currently in use to diagnose LTBI. IFN-γ is known to regulate the synthesis and secretion of MIF [31]. In previous studies, MIF secretion from macrophages was reportedly induced by TNF-α and IFN-γ [32].

Although there are few reports of anti-MIF antibodies to date, anti-IFN-γ antibodies have been previously described in patients with TB, nontuberculous mycobacteria, HIV, and African trypanosomiasis [33,34,35]. Anti-IFN-γ autoantibodies appear to be associated with the inhibition of downstream cytokine induction. Patel et al. reported that autoantibodies against IFN-γ antibodies existed in high-titer in severe nontuberculous mycobacterial infections [35]. In addition, Madariaga et al. demonstrated the presence of anti-IFN-γ autoantibodies in serum from patients with TB [33]. They hypothesized that as the disease progresses, the level of autoantibody production increases, and that the suppression of the Th1-type immune response contributes to the progression of the disease [33].

Autoantibodies against cytokines have been reported in several previous studies, but their physiological and pathophysiological significance is mostly unknown [21]. In some studies, it was assumed that autoantibodies against cytokines regulate biological activity by neutralizing cytokines [36], and act as cytokine reservoirs by extending their half-life via forming cytokine-autoantibody complexes [37]. Therefore, when considering the results of previous studies, we speculate that the antibody response to MIF observed in the current study is likely to be related to the antagonistic effect on MIF expression that is induced via IFN-γ. Previous studies showed that autoantibodies against GM-CSF [38], IL-2 [39], IL-6 [40], and IFN-γ [20] neutralized the respective cytokines in vitro. The levels of anti-IL-6 Ab were quantified in 4,230 blood donors, and high anti-IL-6 Ab titers were associated with IL-6 deficiency in vivo [40]. Additionally, in patients with tuberculosis, low levels of anti-MIF autoantibodies are believed to be associated with increased activation of MIF [40]. In order to clarify this mechanism, it would be necessary to measure the levels of anti-MIF autoantibodies and perform MIF neutralization assays in future studies.

The present study has several limitations. The production of MIF was not measured. In addition, it was not confirmed whether anti-MIF autoantibodies were affected by other cytokines whose levels may increase in TB infection. Thus, further studies are needed in order to confirm the functional relationship between anti-MIF autoantibodies and MIF. 

To the best of our knowledge, this is the first study to analyze responses to MIF in NI, LTBI, and ATB groups. Further, IgA antibody responses to MIF could be a potential target for diagnosing LTBI. Although none of the antibodies could individually achieve high diagnostic predictive power, these findings suggest that antibodies to MIF have the potential to be used in the development of rapid assays that complement existing TB diagnostic methods. 

## Figures and Tables

**Table 1:** Efficiencies of immunoglobulin (Ig)A and IgG against MIF as diagnostic markers for tuberculosis.

Groups and Antibody	AUC (*p* Value)	Cut-Off (OD)	Sensitivity (%)	Specificity (%)
**IgA response to MIF**				
NI vs. LTBI	0.7161 (<0.0001)	0.7825	69.8	75.8
NI vs. ATB	0.6919 (<0.001)	0.8835	69.2	67.7
LTBI vs. ATB	0.6398 (<0.01)	0.7075	64.6	62.3
**IgG response to MIF**				
NI vs. LTBI	0.6397 (<0.05)	1.0175	64.2	59.7

MIF, macrophage migration inhibitory factor; AUC, area under the curve; OD, optical density; NI, non-infected; LTBI, latent tuberculosis infection; ATB, active tuberculosis.
